# Differential Impact of Calcium and Vitamin D on Body Composition Changes in Post-Menopausal Women Following a Restricted Energy Diet and Exercise Program

**DOI:** 10.3390/nu12030713

**Published:** 2020-03-07

**Authors:** Chad M. Kerksick, Michael D. Roberts, Bill I. Campbell, Melyn M. Galbreath, Lemuel W. Taylor, Colin D. Wilborn, Ashli Lee, Jacqueline Dove, Jennifer W. Bunn, Christopher J. Rasmussen, Richard B. Kreider

**Affiliations:** 1 Exercise and Performance Nutrition Laboratory, School of Health Sciences, Lindenwood University, Saint Charles, MO 63301, USA; ckerksick@lindenwood.edu; 2 School of Kinesiology, Auburn University, Auburn, AL 36849, USA; mdr0024@auburn.edu; 3 Edward via College of Osteopathic Medicine, Auburn, AL 36849, USA; 4 Exercise Science Program, University of South Florida, Tampa, FL 33620, USA; bcampbell@usf.edu; 5 Matrix Medical Net, New York, NY 10011, USA; galbreathmelyn@gmail.com; 6 Exercise and Sports Science Department, University of Mary Hardin-Baylor, Belton, TX 76513, USA; ltaylor@umhb.edu (L.W.T.); cwilborn@umhb.edu (C.D.W.); 7 Health, Human Performance, and Recreation Department, Baylor University, Waco, TX 76798, USA; ashli.r.lee@gmail.com; 8 Department of Biology, McClennan Community College, Waco, TX 76798, USA; jdove@mcclennan.edu; 9 Department of Physical Therapy, Campbell University, Buies Creek, NC 27506, USA; bunnj@campbell.edu; 10 Exercise & Sport Nutrition Lab, Human Clinical Research Facility, Department of Health and Kinesiology, Texas A & M University, College Station, TX 77843, USA; crasmussen@tamu.edu

**Keywords:** weight loss, dietary supplement, fat loss, training adaptations

## Abstract

Vitamin D and calcium supplementation have been posited to improve body composition and different formulations of calcium may impact bioavailability. However, data are lacking regarding the combinatorial effects of exercise, diet, and calcium and/or vitamin D supplementation on body composition changes in post-menopausal women. Herein, 128 post-menopausal women (51.3 ± 4.5 years, 36.4 ± 5.7 kg/m^2^, 46.2 ± 4.5% fat) were assigned to diet and supplement groups while participating in a supervised circuit-style resistance-training program (3 d/week) over a 14-week period. Diet groups included: (1) normal diet (CTL), (2) a low-calorie, higher protein diet (LCHP; 1600 kcal/day, 15% carbohydrates, 55% protein, 30% fat), and (3) a low-calorie, higher carbohydrate diet (LCHC; 1600 kcal/day, 55% carbohydrates, 15% protein, 30% fat). Supplement groups consisted of: (1) maltodextrin (PLA), (2) 800 mg/day of calcium carbonate (Ca), and (3) 800 mg/day of calcium citrate and malate and 400 IU/day of vitamin D (Ca+D). Fasting blood samples, body composition, resting energy expenditure, aerobic capacity, muscular strength and endurance measures were assessed. Data were analyzed by mixed factorial ANOVA with repeated measures and presented as mean change from baseline [95% CI]. Exercise training promoted significant improvements in strength, peak aerobic capacity, and blood lipids. Dieting resulted in greater losses of body mass (CTL −0.4 ± 2.4; LCHC −5.1 ± 4.2; LCHP −3.8 ± 4.2 kg) and fat mass (CTL −1.4 ± 1.8; LCHC −3.7 ± 3.7; LCHP −3.4 ± 3.4 kg). When compared to LCHC-PLA, the LCHC + Ca combination led to greater losses in body mass (PLA −4.1 [−6.1, −2.1], Ca −6.4 [−8.1, −4.7], Ca+D −4.4 [−6.4, −2.5] kg). In comparison to LCHC-Ca, the LCHC-Ca+D led to an improved maintenance of fat-free mass (PLA −0.3 [−1.4, 0.7], Ca −1.4 [−2.3, −0.5], Ca+D 0.4 [−0.6, 1.5] kg) and a greater loss of body fat (PLA −2.3 [−3.4, −1.1], Ca −1.3 [−2.3, −0.3], Ca+D −3.6 [−4.8, −2.5]%). Alternatively, no significant differences in weight loss or body composition resulted when adding Ca or Ca+D to the LCHP regimen in comparison to when PLA was added to the LCHP diet. When combined with an energy-restricted, higher carbohydrate diet, adding 800 mg of Ca carbonate stimulated greater body mass loss compared to when a PLA was added. Alternatively, adding Ca+D to the LCHC diet promoted greater% fat changes and attenuation of fat-free mass loss. Our results expand upon current literature regarding the impact of calcium supplementation with dieting and regular exercise. This data highlights that different forms of calcium in combination with an energy restricted, higher carbohydrate diet may trigger changes in body mass or body composition while no impact of calcium supplementation was observed when participants followed an energy restricted, higher protein diet.

## 1. Introduction

The overweight and obesity pandemic poses a global burden, with as many as 1.5 billion people, or roughly 20% of the world population being affected [1] while a 2019 report stating that by the year 2030 50% of U.S. adults will have obesity [2]. In addition, many reports have highlighted the fact that this dilemma poses severe economic consequences [3,4]. In this regard, obese persons bear medical and prescription medicine costs that are approximately 30% greater than normal-weight peers [5] or approximately $2700 USD annually [6]. Furthermore, lost quantity of lifetime in obese persons has been estimated to be as high as 20 years compared to normal-weight peers [7]. Hence, while the World Health Organization reports that life expectancy in developed countries is nearly 80 years old, debilitation resulting from obesity can begin (and rapidly accelerate) as early as the fifth decade of life. Although the causes of obesity stem from environmental and genetic factors, post-menopausal women may be at increased risk due to factors such as reductions in circulating sex hormones [8,9], decrements in whole-body metabolism due to the lack or irregular presence of a menstrual cycle [10] and gender-independent aging mechanisms [11]. Therefore, countermeasures to reduce obesity rates continue to be the focus of much scientific research, particularly in post-menopausal women given their relative susceptibility for developing the disease.

It is well established that exercise [12,13,14,15] and/or engaging in hypocaloric higher-protein/lower-carbohydrate diets [14,16,17] improves body composition in overweight and obese individuals. These reports extend to post-menopausal women, whereby researchers have reported that exercise training [18] and higher protein, lower-carbohydrate dieting can improve body composition [19]. Beyond diet and exercise interventions, supplementing the diet with select micronutrients may be a viable non-pharmaceutical option in augmenting weight loss. For instance, high calcium intakes have been associated with higher levels of fat oxidation [20] and increased satiety [21], while vitamin D signaling has also been reported to favorably affect the expression patterns of lipolytic and thermogenic genes in adipose tissue [22,23]. In multiple reports, Zemel and colleagues [24,25,26] first highlighted the potential impact of calcium and dairy on weight loss, weight gain, and fat loss. In this respect, initial animal work demonstrated attenuations of adipocyte accretion [27] and weight gain while manipulating calcitriol levels has been shown to impact weight gain [26]. Further, diets higher in calcium in transgenic animal work has been shown to impede fat loss [24]. These authors purported that a mechanistic impact of calcium may be linked to various bioactive compounds found in dairy that act synergistically with calcium. Additionally, evidence by Wang et al. [28] suggested that different forms of calcium (citrate vs. malate) have differing levels of bioavailability in healthy, premenopausal women. In a crossover design, identical doses (1000 mg) of calcium carbonate (1000 mg powder) and calcium citrate (2 × 500 mg tablets) were provided and pharmacokinetic patterns of blood calcium levels were compared. Results indicated that a single serving of calcium carbonate powder was more bioavailable than calcium citrate tablets. Interestingly, a previous clinical trial [29] reported that both higher dairy calcium intake and relatively high serum vitamin D were associated with a greater diet-induced weight loss over a two-year intervention, and other evidence also suggests that calcium and vitamin D supplementation can facilitate weight loss [21]. However, others have reported that calcium supplementation does not affect body composition in subjects participating in longer-term weight loss interventions [30,31]. Likewise, a recent meta-analysis suggests that there is equivocal evidence with regards to calcium facilitating weight loss, and there is a lack of evidence with regards to vitamin D supplementation being able to improve body composition [32]. Notwithstanding, the aforementioned investigations differed with regards to dietary strategies for weight loss, and most of the studies did not have participants engaging in concomitant exercise training.

While there have been a plethora of data examining the effects of vitamin D and calcium supplementation on weight loss, the potential combinatorial effects of exercise with certain diet prescriptions in combination with calcium and/or vitamin D supplementation on body composition changes requires additional investigation. Moreover, the combinatorial effects of exercise, dieting, and nutritional supplementation on body composition changes has been less studied in post-menopausal overweight women in general. This study assesses whether supplementing the diet with different types of calcium (with or without vitamin D) affects changes in body composition in women participating in a 14-week popular exercise and weight loss program (no diet versus hypocaloric lower- and higher-carbohydrate diets) in previously sedentary post-menopausal subjects. Notably, the exercise program used in this proposed study consists of a 30-min circuit-style exercise program that targets all major muscle groups using bi-directional resistance training machines. Millions of women using the Curves International exercise program use this program worldwide and it has been used successfully in other research studies to stimulate weight and improve health, aerobic fitness, and musculoskeletal fitness. This In light of previously highlighted data, different forms of calcium were investigated in the current study to identify their potential impact on body mass loss and body composition changes. Further, alterations in macronutrient amounts were utilized to identify any combinatory impact of these different forms of calcium in combination with vitamin D due to the observed potential for diets containing different amounts of macronutrients to impact body composition changes [14,33,34,35]. Thus, we hypothesized that individuals on the lower-carbohydrate diet would experience a greater improvement in body composition compared to those that did not diet and/or those that were assigned to the higher-carbohydrate diet. Moreover, we hypothesized that, irrespective of diet composition, participants supplementing with calcium and vitamin D would experience better improvements in body composition. Secondary objectives of this study were to also examine if the implemented diet and supplementation strategies with exercise affected resting metabolism and fitness status.

## 2. Methods

### 2.1. Experimental Design

This study was conducted in a randomized, parallel arm, prospective manner at a university-based research facility. The randomization was completed in a block format with two times (2:1:1) the people being assigned to LCHP and LCHC to account for anticipated greater attrition in these groups. This research protocol was reviewed and approved by the Baylor University Institutional Review Board (IRB 2008-0480) prior to initiation of the study in accordance with the Declaration of Helsinki [36] and subsequently approved by the Texas A&M Human Participant Protection Board (IRB 2008-0643F) when the principal investigator moved to that institution. The overall study design including the dietary assignment and exercise program were modeled after the Curves for Women exercise program that has been and is still being used by millions of women worldwide. The specific focus of this investigation was to identify the impact of adding different forms of calcium supplementation with and without the addition of vitamin D to energy restricted diets of higher and lower proportions of carbohydrate and protein. The primary outcomes for this investigation were weight loss and body composition changes. Secondary outcomes included markers of fitness and health. This trial was registered at clinicaltrials.gov as NCT03878667.

### 2.2. Participants

**Figure 1:**
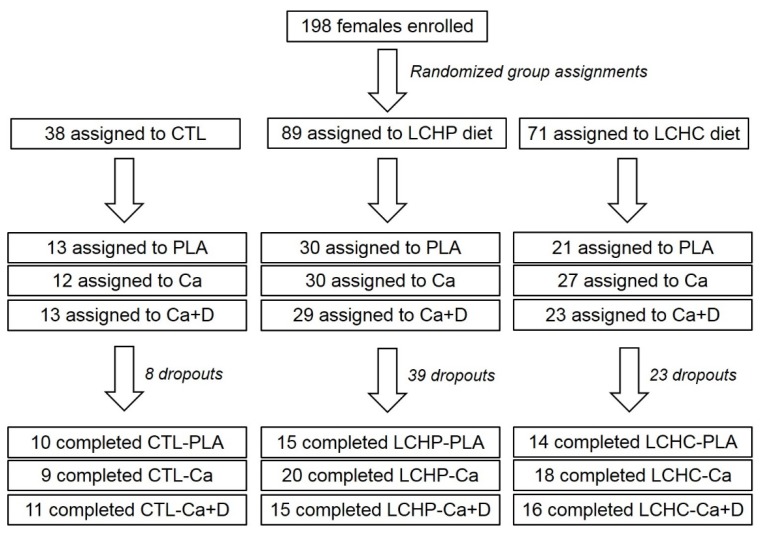
CONSORT diagram. CTL represents the control (no diet, exercise only) group, LCHP represents low calorie—high protein diet, exercise group, LCHC represents low calorie—high carbohydrate, exercise group, PLA represents placebo supplementation, exercise group, Ca represents calcium supplementation, exercise group, Ca+D represents calcium plus Vitamin D supplementation, exercise group.

Participants were recruited through area physicians, advertisements in local newspapers, campus flyers, and internet advertisements. Interested participants were asked to contact the laboratory for an initial telephone pre-screening interview. General entrance criteria included: (a) being an apparently healthy post-menopausal female with a body mass index (BMI) greater than 27 kg/m^2^ and/or body fat percentage above 35%, (b) no recent participation in a diet or exercise program, and (c) capable of engaging in exercise training. Individuals who met the initial entrance criteria were invited to attend a familiarization session in which the details of the study were explained, consent forms were signed, and personal and medical history information was obtained. Thereafter, participants were assigned to their respective supplement and diet group (described below) and were scheduled for baseline (week 0) assessments. Figure 1 presents a CONSORT diagram. A total of 198 women met initial phone screening criteria, consented to participate in the study and underwent baseline testing. Of these, a total of 128 women who were 51.3 ± 4.5 years old, 89.3 ± 15.9 kg, 36.4 ± 5.7 kg/m^2^, and 46.2 ± 4.5% body fat) completed the study. Those who withdrew did so primarily due to time constraints and/or poor compliance to the exercise and diet protocol. Study recruitment spanned approximately 12 months.

### 2.3. Exercise Intervention

All participants engaged in a whole-body circuit training resistance exercise program for 14-weeks. Briefly, participants performed the 30-min Curves circuit exercise program (Curves International, Waco, TX, USA) three days per week over the course of the 14-week study. The circuit included 13 bi-directional hydraulic concentric only resistance exercise machines which emphasized all major muscle groups (i.e., elbow flexion/extension, knee flexion/extension, shoulder press/lat pull, hip abductor/adductor, chest press/seated row, horizontal leg press, squat, abdominal crunch/back extension, chest flies, oblique, shoulder shrug/hip, hip extension, and side bends). During each training session, participants were coached to perform as many repetitions as possible within a 30-s time period on each resistance machine. Between machines, participants performed floor-based exercises (e.g., stepping, calisthenics, etc.) for 30-s with a goal of maintaining an elevated heart rate. Thus, each participant completed 30-s on a machine, followed by 30-s of floor-based calisthenics until they completed two complete circuits (26 stations total). A brief period of stretching was completed following the completion of their exercise circuit. Previous work from our lab indicated this workout regimen yields an average exercise heart rate of 126 ± 15 bpm (80% of maximal heart rate), exercise intensity of 65 ± 10% of peak oxygen uptake, resistance exercise intensities ranging between 61% and 82% of 1RM, and expends approximately 314 ± 102 kcals per workout [37,38,39]. All workouts were supervised by trained fitness instructors who monitored proper exercise technique and maintenance of adequate exercise intensity. Compliance to the exercise program was set a priori at a minimum of 70% compliance (30/42 exercise sessions).

### 2.4. Diet Intervention

As consistent with several previously published studies [14,15,40,41,42,43,44] from our research group over the past ten years and has encompassed over 750 research participants, participants in this study were assigned at baseline in a block fashion according to age and body mass: (1) No dietary modification (CTL); (2) A low-calorie, higher-protein (LCHP) diet consisting of 15% carbohydrates, 55% protein, and 30% fat); or, (3) A low-calorie, higher-carbohydrate (LCHC) diet consisting of 55% carbohydrates, 15% protein, and 30% fat). Across the entire 14-week study protocol, the LCHP and LCHC diets consisted of 1200 kcal/day for 1 week and 1600 kcal/day for 9 weeks. For the remaining four weeks, participants in these groups were instructed to weigh themselves daily while ingesting a 2600 kcal/day diet (55% carbohydrates, 15% protein, 30% fat). When three pounds (~1.5 kg) were gained, participants were instructed to follow a 1200 kcal/day until the gained weight was lost. Previous research has demonstrated that this 14-week program promotes a 3–5 kg weight loss while maintaining resting energy expenditure in sedentary overweight pre-menopausal women [12,13,27,28,29,30,31]. Of note, participants were given diet plans and menus to follow at the start of the study. Participants also met with a registered dietitian at each testing session to discuss diet compliance.

### 2.5. Supplementation Protocol

As previously observed by Wang et al. [28] assessing the bioavailability and therefore potential differential impact on weight loss while following higher or lower carbohydrate diets throughout an exercise program was the key rationale for using different forms of calcium. Towards this aim, participants were assigned to ingest in a randomized and double-blinded manner dietary supplements containing total doses of: (1) 800 mg of a maltodextrin placebo (PLA); (2) 800 mg of calcium as carbonate (Ca); or, (3) 800 mg of calcium as a combination of citrate tetrahydrate and citrate malate, 400 IU/day of vitamin D as cholecalciferol, 300 mg of magnesium as magnesium oxide, 7.5 mg of zinc as zinc gluconate, 2 mg of copper as hydrolyzed protein chelate and 2 mg of manganese as manganese gluconate (Ca+D). Each participant’s total dose was evenly divided into two equal daily doses. Participants were instructed to consume their assigned dosage 30 min before eating in the morning and again in the evening before an evening meal for 14 weeks. Supplements were prepared by Nutra™ Manufacturing (Greenville, SC, USA) and prepared in generic and coded bottles for double blinded administration. Supplementation compliance was monitored by having the participants return empty bottles of the supplement at the end of each testing phase. In addition, internal monitoring of supplementation compliance occurred with participants completing a compliance statement in a post-study questionnaire.

## 3. Procedures

### 3.1. Dietary Assessment

Dietary food logs (3 weekdays and 1 weekend day) were filled out by participants during the pre-intervention (baseline), week 1 into the intervention (week 1) and one-week prior to the post-testing period (week 14). Logs were reviewed by a registered dietitian and subsequently analyzed for average energy and macronutrient intake using the ESHA Food Processor v8.6 Nutritional Analysis software (ESHA Research Inc., Salem, OR, USA).

### 3.2. Resting Energy Expenditure and Metabolism

Resting energy expenditure (REE) and respiratory exchange ratio (RER) were assessed using a ParvoMedics TrueMax 2400 Metabolic Measurement System (ParvoMedics, Inc., Sandy, UT, USA). These tests were performed with the participants lying supine on an exam table for approximately 20 min. A dilution pump controlled the flow of air to ensure a consistent percentage of carbon dioxide (0.8–1.2%) flowing through the metabolic cart. Data were analyzed after the first 10 min of testing during a five-minute period of time in which criterion variables (e.g., O_2_ uptake and CO_2_ expiration) were within a 5% range. REE assessments in a previously published study from our laboratory [14] revealed that the coefficient of variation for REE ranged from 8.2–12.0% with a mean intra-class coefficient of 0.942.

### 3.3. Body Weight and Composition

Height and body mass were determined according to standard procedures using a calibrated electronic scale (Cardinal Detecto Scale Model 8430, Webb City, MO, USA) with a precision of ±0.02 kg. Body composition (excluding cranium) was assessed using a Hologic Discovery W (Hologic Inc., Waltham, MA, USA) DXA equipped with APEX Software (APEX Corporation Software, Pittsburg, PA, USA). Test-retest reliability studies performed with this DXA machine have previously yielded mean coefficients of variation for bone mineral content and lean mass of 0.31–0.45% with a mean intra-class correlation of 0.985 [44].

### 3.4. Resting Hemodynamics and Exercise Capacity

Resting heart rate was determined by palpation of the radial artery using standard procedures [45]. Blood pressure was assessed by auscultation of the brachial artery using an aneroid sphygmomanometer using standard clinical procedures [45]. Resting heart rate and blood pressure measurements were taken on the participant in the supine position after resting for 5 min. Participants were attached to a Quinton 710 ECG (Quinton Instruments, Bothell, WA, USA) and walked on a Trackmaster TMX425C treadmill (JAS Fitness Systems, Newton, KS, USA). Expired gases were collected using a Parvo Medics 2400 TrueMax Metabolic Measurement System (ParvoMedics, Inc., Sandy, UT, USA). Participants then performed a standard symptom-limited treadmill exercise test following the Bruce protocol to maximal exertion. Standard test termination criteria according to the American College of Sports Medicine were applied [45]. Calibration of gas and flow sensors was completed every morning prior to testing and was found to be within 3% of the previous calibration point.

### 3.5. Muscular Strength and Endurance

A standard isotonic Olympic bench press (Nebula Fitness, Versailles, OH, USA) was used for the isotonic bench press testing. A 1RM testing procedure was performed using standard procedures with 2-min recovery between attempts [45]. Following 1RM testing, participants performed a maximum number of repetitions test at 80% of 1RM using the bench press exercise to determine upper body muscular endurance. Participants were then given five minutes of rest and completed identical 1RM and maximal repetitions to fatigue tests using a hip sled/leg press (Nebula Fitness, Versailles, OH, USA) [45]. Test to test reliability of performing these strength tests in our lab has yielded low mean coefficients of variation and high reliability for the bench press (CV: 1.9%, intra-class *r* = 0.94) and hip sled/leg press (CV: 0.7%, intra-class *r* = 0.91).

### 3.6. Blood Collection and Analysis

Fasted whole blood and serum samples were collected using standard phlebotomy techniques. Whole blood samples were analyzed for complete blood counts with platelet differentials using an Abbott Cell Dyn 3500 (Abbott Laboratories, Abbott Park, IL, USA) automated hematology analyzer. Serum samples were analyzed for a complete metabolic panel using a calibrated Dade Behring Dimension RXL (Siemens AG, Munich, Germany) automated clinical chemistry analyzer. Coefficient of variation (CV) for the tests using this analyzer was similar to previously published data for these tests (range: 1.0 to 9.6%) [46].

### 3.7. Statistical Analysis

A priori power calculation was set at >0.80 and was based on the observed change in fat mass between diet groups from previous research in our lab utilizing similar diet and exercise interventions [14,15,40,42,43,44,47]. This analysis revealed that a sample size of 15–20 participants per group was sufficient to detect meaningful changes (~2 kg) in fat mass between the hypoenergetic diet (LC) groups. All data were analyzed with IBM^®^ SPSS^®^ Version 25 software (IBM Corp., Armonk, NY, USA). General linear modal (GLM) multivariate analysis was used to determine differences between groups at baseline. Related variables were analyzed using univariate, multivariate and repeated measures GLM. The overall multivariate Wilks’ Lambda and Greenhouse-Geisser univariate p-levels are reported. Data were considered significant when the probability of type I error was 0.05 or less and statistical trends toward significance were noted if the p-level ranged between 0.05 and 0.10. Partial eta squared effect sizes (η_p_^2^) are reported when statistical trends toward significance was observed as an indicator of effect size [48]. An eta squared around 0.02 was considered small, 0.13 medium, and 0.26 large [48]. Tukey’s least significant differences (LSD) post-hoc analyses were performed to determine differences among groups. Mean changes from baseline as well as percent changes from baseline were calculated and analyzed using one-way ANOVA to determine mean changes with 95% confidence intervals (CI). Mean changes with 95% CI’s completely above or below baseline were considered significantly different [48]. Data are presented as means ± standard deviations, mean [95% CI] change from baseline, or mean [95% CI] percent change from baseline.

## 4. Results

### 4.1. Baseline Characteristics

Baseline participant demographics are presented in Supplemental Table S1. Multivariate GLM analysis revealed overall Wilks’ Lambda diet (*p* = 0.295), supplement (*p* = 0.886) and diet x supplement (*p* = 0.885) effects. Significant diet effects were observed among groups in absolute resting energy expenditure expressed in kcal/day (*p* = 0.01) but not relative energy expenditure expressed in kcal/kg/day (*p* = 0.14). No significant differences were observed among supplemented groups.

### 4.2. Energy and Macronutrient Intake

Self-reported energy and macronutrient intakes are presented in Supplemental Table S2. Overall GLM multivariate analysis revealed Wilks’ Lambda time (*p* < 0.001), time × diet (*p* < 0.001), time x supplement (*p* = 0.130), and time x diet x supplement (*p* = 0.410) effects. Univariate analysis revealed that energy intake was significantly reduced by 517 (95% CI: 354,681) kcals/day during the 10-week weight loss phase of the intervention and 276 (95% CI: 107,445) kcals/day during the maintenance period. Carbohydrate intake was significantly lower while protein intake was significantly higher in the LCHP group. Moreover, participants in the LCHC were able to reduce fat intake to a greater degree. Thus, the dietary intervention effectively altered energy and macronutrient intake.

### 4.3. Resting Energy Expenditure and Metabolism

Supplemental Table S3 presents resting energy expenditure results. The overall GLM multivariate analysis revealed Wilks’ Lambda time (*p* < 0.001), time × diet (*p* < 0.001), time × supplement (*p* = 0.854), and time x diet x supplement (*p* = 0.096) effects. Univariate analysis revealed that although absolute REE was significantly decreased by about 80 kcal/day on average over time, no significant time effects were observed in relative REE. Moreover, absolute REE decreased to a greater degree in the Ca+D group during the weight loss intervention but this was not significantly different when expressed relative to body weight. Respiratory exchange ratio also decreased over time indicative of greater fat oxidation with some evidence that RER decreased from baseline to a greater degree in the LCHC group.

### 4.4. Body Weight and Composition

Body weight and composition results are presented in Supplemental Table S4. GLM multivariate analysis of body weight, fat mass, fat free mass, and body fat revealed Wilks’ Lambda time (*p* < 0.001), time × diet (*p* < 0.001), time x supplement (*p* = 0.799), and time x diet x supplement (*p* = 0.507) effects. Participants in the LCHC and LCHP groups experienced significantly greater fat loss (*p* = 0.008) and reduction in percent body fat (*p* < 0.001) over time with no significant differences observed between the LCHC and LCHP groups. Meanwhile, fat free mass significantly increased in the exercise only control group while being maintained in the diet groups. No significant time x supplement effects were observed in weight (*p* = 0.552), fat mass (*p* = 0.708), fat free mass (*p* = 0.517), or percent body fat (*p* = 0.412) although a time × supplement × diet trend was observed in percent body fat (*p* = 0.08). Post-hoc analysis revealed that participants following the LCHC diet and supplementing the diet with Ca+D experienced greater fat loss than those supplementing the diet with Ca (*p* = 0.018). A significant time × supplement interaction was also observed in bone mineral content (*p* = 0.026). However, post-hoc analysis did not reveal any significant differences among groups.

**Figure 2:**
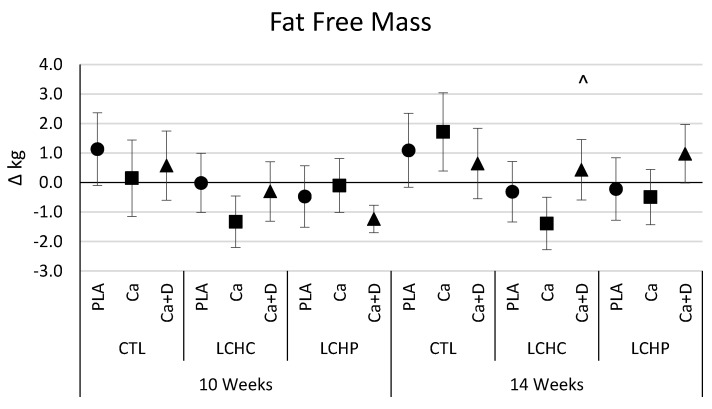

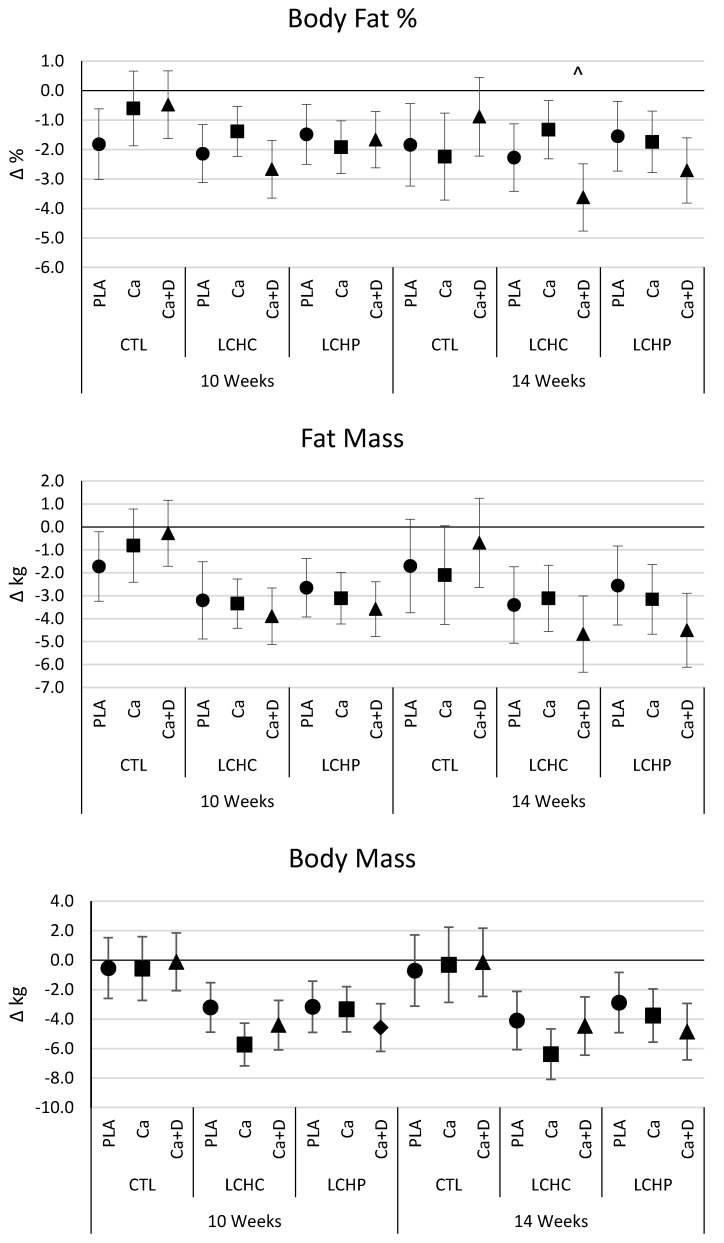
Body mass and composition changes from baseline (mean [95% CI]) observed for the placebo (PLA or ●), calcium (Ca or ■), and calcium + Vitamin D (Ca+D or ▲) groups. CTL represents the control (no diet, exercise only) group, LCHP represents low calorie—high protein diet, exercise group, LCHC represents low calorie—high carbohydrate, exercise group, PLA represents placebo supplementation, exercise group, Ca represents calcium supplementation, exercise group, Ca+D represents calcium plus Vitamin D supplementation, exercise group.† represents *p* < 0.05 difference between PLA and Ca groups. ^ represents *p* < 0.05 difference between Ca and Ca+D groups.

Figure 2 presents mean changes from baseline with 95% CI in body composition variables. Participants adhering to the LCHC and LCHP diets observed significant within-group reductions in body mass, fat mass, and body fat% after 10 and 14 weeks of dieting. Changes in body composition were effectively maintained during the 4-week maintenance period. Participants in the LCHC-Ca group experienced greater weight loss than the LCHC-PLA group after 10 and 14 weeks of interventions while no differences were observed between the LCHC-Ca+D (and LCHC-PLA) groups at these respective timepoints. Additionally, participants in the LCHC-Ca+D group reported greater fat-free mass maintenance and a greater reduction in percent body fat after 14 weeks in comparison to changes seen in LCHC-Ca. No differences in fat-free mass or body fat mass were noted for the LCHC-PLA group. While participants in the LCHP lost body weight and observed significant reductions in percent body fat, supplementation had no significant effects on these responses.

### 4.5. Resting Hemodynamics and Aerobic Capacity

Supplemental Table S5 presents resting hemodynamic and aerobic capacity data. The overall GLM multivariate analysis revealed Wilks’ Lambda time (*p* < 0.001), time x diet (*p* < 0.001), time × supplement (*p* = 0.349), and time × diet × supplement (*p* = 0.862) effects. Univariate analysis revealed no significant changes in resting heart rate over time or among diet and supplement groups. Systolic blood pressure decreased over time (−4.5 ± 12 bpm, *p* = 0.001) with no time x diet (*p* = 0.204) differences observed while a trend was observed in time x supplement effects (*p* = 0.055). Post-hoc analysis revealed that resting SBP was significantly lower in the Ca+D group at baseline and after 10-weeks in comparison to the PLA group. Diastolic blood pressure also decreased from baseline in all groups (−4.0 [−6.3, −1.7]%) with no significant time x diet (*p* = 0.322), time x supplement (*p* = 0.666), or time × diet × supplement (*p* = 0.661) effects. Relative peak oxygen uptake increased over time by 6.1 [2.9, 9.4]% with no significant differences observed among diet or supplement groups.

### 4.6. Muscular Strength and Endurance

Supplemental Table S6 shows upper and lower extremity muscular strength and endurance results. Overall GLM multivariate analysis revealed Wilks’ Lambda time (*p* < 0.001), time × diet (*p* < 0.667), time × supplement (*p* = 0.451), and time x diet x supplement (*p* = 0.557) effects. One repetition maximum (1RM) bench press increased by 3.0 [1.8, 4.2]% while 1RM leg press increased by 13.0 [7.1,19.0]% over time. However, no significant time × diet, time × supplement, or time × diet × supplement effect were observed.

### 4.7. Blood Glucose and Lipids

Supplemental Table S7 presents blood glucose and lipid results observed. The overall GLM multivariate analysis revealed Wilks’ Lambda time (*p* = 0.001), time × diet (*p* < 0.023), time × supplement (*p* = 0.472), and time x diet x supplement (*p* = 0.975) effects. Univariate analysis revealed significant time effects in total cholesterol, high-density lipoprotein, and low-density lipoprotein values with no significant differences observed among diet or supplement groups. A significant time × diet effect was observed in serum triglycerides with participants following the LCHC diet having lower triglyceride values than the CTL group throughout the study and participants in the LCHP group experiencing lower triglycerides after 10-weeks of intervention.

## 5. Discussion and Conclusions

The present study provides unique data examining the potential combinatorial effects of exercise and different diets with different forms of calcium with and without vitamin D supplementation on body composition changes in post-menopausal women that participated in an exercise program. The primary findings reveal that the LCHC and LCHP diets promoted statistically significant decreases in body and fat mass from weeks 0 to 14, whereas the CTL diet did not. Moreover, these findings also indicate that calcium supplementation may operate most effectively to induce favorable changes in body composition when combined with a hypoenergetic diet that contains a greater proportion of carbohydrate versus protein. However, contrary to our preliminary hypothesis, there were no main supplement effects or diet x supplement interactions with regards to changes in body composition. These findings are discussed in greater detail below.

### 5.1. Primary Outcome—Weight Loss and Body Composition

Calcium and vitamin D consumption have been widely investigated with regards to their effects on body composition. Shahar et al. [29] reported that higher calcium intake and relatively high serum vitamin D levels were associated with a greater diet-induced weight loss over a two-year intervention. Major et al. [21] similarly reported that low-calcium consumers experienced a significant decrease in fat mass when supplementing with calcium and vitamin D. Similar to our findings, however, larger-scale investigations have failed to demonstrate that calcium supplementation promotes weight loss. For instance, Shapses et al. [31], who combined data from three 25-week placebo-controlled trials that administered 1000 mg/day calcium supplementation and modest caloric restriction in 100 premenopausal and postmenopausal women, reported that there were no significant differences between placebo and calcium-supplemented participants in body weight (Ca: −7.0 kg, placebo: −6.2 kg) or fat mass changes (Ca: −5.5 kg, placebo: −4.5 kg). Wagner et al. [30] also examined the effects of a 12-week dietary intervention (500 kcal restriction) with exercise (3 d/week) and 800 mg/day of calcium supplementation (Ca lactate vs. Ca phosphate vs. milk) in pre-menopausal women and reported weight loss was similar between the placebo and experimental groups (Ca lactate: 4.1 kg, Ca phosphate: 5.4 kg, milk: 4.2 kg, placebo: 5.8 kg). Thus, our data expands upon current literature to suggest that calcium supplementation with dieting and regular exercise does not further improve changes in body mass or body composition in post-menopausal women.

We also report that vitamin D supplementation when combined with calcium supplementation and energy-restricted diets of varying macronutrient ratios, may exert differing influences on body composition. Previous investigations somewhat conflict with this outcome. For example, Zittermann et al. [49] reported that 12 months of vitamin D supplementation (3320 IU/day) and dieting did not significantly affect weight loss in overweight subjects (vitamin D: −5.7 kg, placebo −6.4 kg). Our data also partially align with a recent study reporting that overweight/obese women (50–75 year of age) supplementing with 2000 IU/day of vitamin D while on a reduced-calorie diet and participating in 225 min/week of aerobic activity lost a similar amount of body mass and percent body fat over a 12-month period compared to those that supplemented with a placebo (body mass: vitamin D −7.1 kg, placebo −7.4 kg; percent body fat: vitamin D −4.1%, placebo −3.5%). Both of these studies, however, did not combine vitamin D with calcium or changed the macronutrient distribution and notably, each of these diets supplemented with higher amounts of vitamin D when compared to the present study. Interestingly, Vimaleswaran et al. [50] performed a series of regression analyses from polymorphisms, serum vitamin D levels and body mass index (BMI) of 42,024 participants and concluded that a higher BMI is associated with lower serum vitamin D levels. While this association may create justification for supplementing with vitamin D to increase serum levels, it was concluded that the potential impact of changing BMI as a result of increasing vitamin D levels was likely to be small. Therein, while our results are similar, we did not calculate vitamin D levels at any point in our intervention, which prevents us from drawing conclusions surrounding any potential relationships between vitamin D levels and changes in our measured outcomes. Our data does clearly highlight that vitamin D supplementation does not improve body composition in females who have also incorporated dieting and/or exercise as part of their weight loss strategy. Moreover, rather than lower vitamin D levels being a contributor to increased body weight/body fat, becoming overweight or obese may decrease vitamin D levels, but these are areas of inquiry in need of more research.

As mentioned previously, high calcium intakes have been associated with higher levels of fat oxidation [20], whereas vitamin D signaling has been associated with increased thermogenic mechanisms [22,23]. While we did not implement mechanistic measures in this study, there was not a supplement effect for changes in REE or RER. Hence, this finding suggests that, while there are metabolic associations with calcium and vitamin D, additional supplementation with these micronutrients at the doses administered herein may not further enhance these metabolic processes.

It should be noted that while Ca and Ca+D exerted no significant differences in body composition between the LCHC and LCHP groups, changes within just the LCHC group revealed that the addition of Ca+D instigated a significant loss of fat mass while no such change was reported in the LCHC or LCHC + Ca groups. This finding provides support that Ca+D supplementation may be more effective for promoting fat loss while following a LCHC diet as compared to higher protein diets. These findings agree with Major and colleagues [51] and in contrast to the findings of Holecki et al. [52] in relation to fat mass loss. Holecki et al. [52] reported that Ca+D supplementation during a 3-month low calorie balanced diet resulted in no additional effect on weight loss or fat loss in obese females. Additionally, no changes in serum levels of vitamin D (as 25-(OH)-D3), parathormone, or calcium were found after the intervention. Major and co-workers [51] reported that in overweight and obese females with very low calcium intakes (<800 mg calcium per day), a significant decrease in fat mass was achieved when supplementing with calcium and vitamin D (as compared to a placebo) during a weight loss program. Clearly, methodological differences among these investigations with the present study could account for the discrepancies in outcomes. In particular, differences in the different forms of calcium supplementation, length of intervention, and inclusion of exercise (among others) likely contribute to the variability in weight loss outcomes reported. In light of this, more research should evaluate the effects of different forms of calcium supplementation on weight loss and body composition changes while adhering to different types of diets and exercise programs.

### 5.2. Secondary Outcomes—Markers of Health & Fitness

As a secondary aim, we sought to examine if calcium alone or calcium plus vitamin D supplementation in combination with a weekly exercise program and two different restricted energy dietary programs (higher carbohydrate and higher protein) augmented exercise adaptations. There were spurious week 0 to 14 changes in select fitness variables within groups, although no consistent supplement effects on the changes in strength and aerobic variables were present. Indeed, there have been no consistent relationships established in the literature regarding calcium supplementation with strength or aerobic fitness, nor have there been reports linking vitamin D supplementation with changes in aerobic fitness. Hence, our findings are not surprising in this regard. Reports do exist, however, suggesting that a low vitamin D status is linked with lower strength and muscle mass levels [53,54,55], however, our study protocol lacked biochemical assessments of vitamin D and thus we were not able to explore these relationships. Despite this association, a recent meta-analysis examining 17 clinical trials (5072 participants) reported that vitamin D supplementation does not have a significant effect on muscle strength, although a limited number of studies demonstrate an increase in muscle strength in adults with vitamin D deficiency [56]. Thus, if most participants in the current study were not vitamin D deficient, these data continue to suggest that vitamin D supplementation in general does not augment muscle strength.

Although we report that calcium alone or calcium plus vitamin D supplementation do not affect body composition, our data clearly demonstrate that modest caloric restriction with the employed exercise program is more effective than exercise alone in reducing fat mass. These fat loss changes are in agreement with our previous findings involving females with a similar exercise program and various dietary assignments [14,15,40,43,44]. Furthermore, numerous studies have reported that hypocaloric diets with exercise can improve body composition in younger and older females over a 12–20+ week period, whereas resistance exercise is typically beneficial in accruing lean body mass, attenuating the loss of fat-free mass and/or maintaining body composition [57,58].

Contrary to our preliminary hypothesis, however, the LCHC and LCHP diets promoted similar weight and fat mass losses in the current study. In this regard, several studies have suggested that lower-carbohydrate diets elicit more favorable changes in body composition compared to higher-carbohydrate diets [59,60,61,62] and this may be due to chronically reducing systemic insulin levels which, in turn, reduces adipogenic and lipogenic mechanisms [63]. However, there is also data to suggest that lower- and higher-carbohydrate hypocaloric diets elicit similar body composition changes. For instance, one study reported that modest calorie restriction was just as effective as a hypocaloric low-carbohydrate diet in facilitating weight loss over a one-year period (−3.0 kg vs. −2.1 kg, respectively; *p* > 0.05) [64]. A similar study reported that a hypocaloric low-carbohydrate/high-fat diet was just as effective at eliciting weight loss in obese participants over a 24-week period compared to a hypocaloric low-fat/high-carbohydrate diet (−11.9 kg vs. −10.1 kg, respectively; *p* > 0.05) [65]. A meta-analysis examining 14 studies ranging from 3–24 months in duration determined that calorie restriction is effective at reducing body weight regardless of whether the experimental diets were lower- or higher-carbohydrate diets [66]. Finally, we recently examined our own data from eight trials and determined that hypocaloric diets that were lower and higher in carbohydrate content promoted similar improvements in weight loss and metabolic syndrome scores [47]. Therefore, while it is widely debated as to whether or not low- versus high-carbohydrate diets promote greater weight loss, our data suggests that the lower- and higher-carbohydrate hypocaloric diets examined herein were equally effective at promoting weight and fat mass losses in overweight post-menopausal females that engage in exercise training. Our findings also suggest that when following a hypocaloric diet with a higher proportion of carbohydrate, supplementation with calcium and vitamin D may support a greater maintenance of fat-free mass tissue and greater losses in body fat percentage.

### 5.3. Limitations

Indeed our study possesses various limitations. Specifically, this study was only 14 weeks in duration and this is a relatively short period of time to observe longer-term success of dietary and exercise interventions. Second, while we enrolled a large number of participants, many participants did not finish the intervention and this led to some groups containing a limited number of participants. Notably, the reported attrition in our study protocol may have been due to the macronutrient assignments made as part of the groups assigned to the LCHP groups as has been reported elsewhere [14] or just the well-known attrition associated exercise and weight loss interventions. Third, we did not assess the calcium or vitamin D status of the participants. In this regard, individuals that may have responded well in these supplement groups could have been deficient in these micronutrients; an effect which we are unable to establish with our current data. In this respect, while our hypotheses involving the C + D groups centered upon the impact of added vitamin D + calcium, other minerals were also included in this complex that could have contributed to our outcomes, however, much more research is needed in this respect.

### 5.4. Conclusions

We report that calcium and vitamin D appear to have no added benefit to dietary and exercise effects on body composition at the provided dosages of 800 mg/day of calcium and 800 mg/day of calcium plus 400 IU/day of vitamin D. Thus, adding calcium or a combination of calcium and vitamin D does not stimulate any greater body composition changes. Conclusions from this study also indicated that calcium supplementation may operate most effectively to induce favorable changes in body composition when combined with a hypoenergetic diet that contains a greater proportion of carbohydrate versus protein. Finally, hypoenergetic diets of varying macronutrient ratios stimulate favorable changes in body mass, fat mass, and body fat percentage. Importantly, the reader should understand that other forms of exercise may have stimulated different changes in our measured outcomes and that our findings are limited to following a modest circuit-style resistance-based program in combination with different dietary regimens. Notwithstanding, this study provides valuable data combining exercise, dietary, and nutritional supplementation approaches in post-menopausal women and continues to suggest that shorter-term supplementation with calcium and/or vitamin D has minimal impact on body composition changes or other exercise adaptations. The impact of longer supplementation regimens and its potential impact on our measured outcomes requires additional research.

## Supplementary Materials

The following are available online at https://www.mdpi.com/2072-6643/12/3/713/s1, Table S1: Participant demographics at baseline, Table S2: Absolute energy and macronutrient intake, Table S3: Resting energy expenditure, Table S4: Body composition, Table S5: Resting Hemodynamics and aerobic capacity, Table S6: Muscular strength and endurance, Table S7: Serum glucose and lipids.
